# Inhibition of Indoleamine-2,3-dioxygenase (IDO) in Glioblastoma Cells by Oncolytic Herpes Simplex Virus

**DOI:** 10.1155/2012/815465

**Published:** 2012-08-13

**Authors:** Bonnie Reinhart, Lucia Mazzacurati, Adriana Forero, Chang-Sook Hong, Junichi Eguchi, Hideho Okada, Wendy Fellows, Ajay Niranjan, Justus B. Cohen, Joseph C. Glorioso, Paola Grandi

**Affiliations:** ^1^Department of Microbiology and Molecular Genetics, School of Medicine, University of Pittsburgh, Pittsburgh, PA 15261, USA; ^2^Department of Human Genetics, School of Public Health, University of Pittsburgh, Pittsburgh, PA 15261, USA; ^3^Department of Neurological Surgery, School of Medicine, University of Pittsburgh, Pittsburgh, PA 15261, USA

## Abstract

Successful oncolytic virus treatment of malignant glioblastoma multiforme depends on widespread tumor-specific lytic virus replication and escape from mitigating innate immune responses to infection. Here we characterize a new HSV vector, JD0G, that is deleted for ICP0 and the joint sequences separating the unique long and short elements of the viral genome. We observed that JD0G replication was enhanced in certain glioblastoma cell lines compared to HEL cells, suggesting that a vector backbone deleted for ICP0 may be useful for treatment of glioblastoma. The innate immune response to virus infection can potentially impede oncolytic vector replication in human tumors. Indoleamine-2,3-dioxygenase (IDO) is expressed in response to interferon **γ** (IFN**γ**) and has been linked to both antiviral functions and to the immune escape of tumor cells. We observed that IFN**γ** treatment of human glioblastoma cells induced the expression of IDO and that this expression was quelled by infection with both wild-type and JD0G viruses. The role of IDO in inhibiting virus replication and the connection of this protein to the escape of tumor cells from immune surveillance suggest that IDO downregulation by HSV infection may enhance the oncolytic activity of vectors such as JD0G.

## 1. Introduction 

Glioblastoma multiforme (GBM) is the most common type of primary brain tumor with an incidence rate of approximately 3 cases per 100,000 people per year in the United States (2004-2005, http://www.cbtrus.org/). The current treatment modality for GBM typically involves surgery to remove the tumor, followed by radiotherapy and adjuvant chemotherapy with temozolomide [1]. The infiltrative nature of the tumor makes complete surgical removal difficult, and tumor recurrence is common at the tumor margin. The median survival for patients with GBM is generally less than two years despite treatment. Clearly, new and effective therapies are needed.

Oncolytic vectors (OVs), viruses that have been designed to undergo lytic replication specifically in tumor cells, provide a novel therapeutic approach to treatment of GBM. Herpes simplex virus-based oncolytic vectors (oHSV) have been used in early-phase clinical trials for a variety of tumors including recurrent GBM. Recent trials using the genetically engineered HSV strains G207, HSV1716, OncoVEX^GM-CSF^ and NV1020 have been encouraging, demonstrating that these viruses are relatively nontoxic for normal tissue and showing instances of promising patient responses [2–6]. However, despite their antitumor potential, the overall effectiveness of these vectors has been limited. This observation warrants the exploration of other mutant vector backbones that may improve tumor cell-dependent vector replication.

The engineering of an oHSV vector relies upon the deletion of viral genes that are needed to efficiently complete the virus life cycle in normal cells, but that are complemented by genetic alterations that commonly occur in tumor cells. The ICP0 protein is an attractive target for the design of an oncolytic vector. ICP0 is a nonessential, immediate early gene product that performs a wide range of functions important for virus replication such as counteracting the IFN response [7–9], maintaining the viral genome in a transcriptionally active state [10–12], and targeting specific cellular proteins for degradation by the proteasome, including proteins important in modulating DNA repair pathways, apoptosis and cell-cycle progression [13–19]. Importantly, mutants lacking ICP0 protein are impaired for growth at low multiplicity of infection (MOI) in most cell lines [20], while vigorous replication has been observed in tumor cell lines such as U2OS osteosarcoma cells [21, 22]. It has recently been demonstrated that other tumor cell lines enhance the replication of ICP0-deficient vectors, and an HSV-1 mutant lacking ICP0 protein expression was shown to increase survival in a mouse breast adenocarcinoma model [23, 24]. The tumor cell-specific replication of ICP0-deficient HSV suggests that this mutant backbone may represent a new class of oHSV vector useful for treatment of GBM.

The innate immune response to virus infection can potentially inhibit virus replication and spread. For example, natural killer (NK) cells recruited to the site of HSV infection release interferon *γ* (IFN*γ*) a cytokine that triggers multiple downstream pathways that can inhibit virus replication [25]. The production of IFN*γ* has been shown to inhibit replication of certain oHSV *in vitro* and virus gene expression *in vivo* [25, 26]. Local production of IFN*γ* induces the synthesis of indoleamine-2,3-dioxygenase (IDO), an enzyme that catalyzes the degradation of the essential amino-acid tryptophan producing kynurenine and multiple downstream metabolites. The consequent depletion of tryptophan from the cell and its surroundings impedes virus replication [27]. Furthermore, kynurenine along with other catabolites has been linked to the escape of tumor cells from immune surveillance [28–30]. Thus, IDO may present obstacles to both OV replication and immune clearance of tumor cells.

In this report, we characterized an ICP0-deficient oHSV vector, JD0G [31], for its ability to replicate in glioblastoma cells in the absence and presence of IFN*γ*. We show that JD0G virus replication was enhanced in glioblastoma cell lines compared to HEL cells, suggesting that the former complement the loss of ICP0 protein. Both wild-type KOS virus and JD0G were moderately sensitive to IFN*γ* at low MOI in glioblastoma cells, an effect that was dramatically reduced at higher MOI. Moreover, while glioblastoma cell lines produced IDO protein when stimulated with IFN*γ*, IDO induction had a minimal effect on virus replication. This observation may be related to our finding that IFN*γ*-induced IDO levels were substantially reduced in glioma cells infected with KOS or JD0G

## 2. Results

### 2.1. JD0G Structure

**Figure 1:**
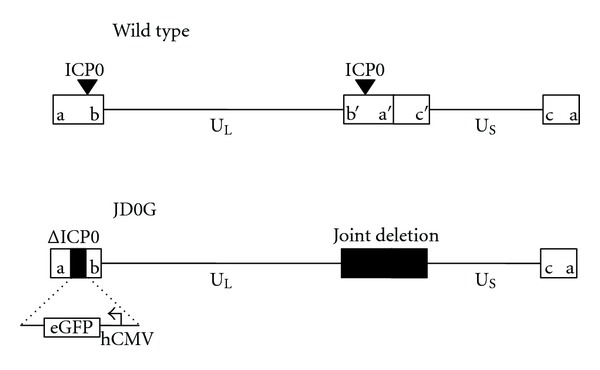
Structure of the JD0G virus compared to the wild-type HSV-1 virus. Schematics depicting the genome structure of wild-type HSV-1 (top) and the JD0G virus (bottom). The unique long (U_L_) and unique short (U_S_) portions of the viral genome are indicated with solid lines, and the repeat regions of the genome (ab/b′a′, a′c′/ca) are shown as open boxes. The locations of the two ICP0 loci are designated with inverted triangles. Black boxes represent deleted regions of the JD0G genome. The joint deletion removes a region spanning positions from 116,982 to 132,605 of the HSV genome (nucleotide positions based on NC_001806). The terminal ICP0-deletion (ΔICP0) replaces the ICP0 coding sequence with an hCMV:eGFP expression cassette.

To create JD0G, the internal repeat sequences “joint” were deleted from the strain KOS HSV-1 genome, a modification that removes one copy each of the gamma 34.5 gene and the immediate early genes encoding ICP0 and ICP4. The removal of the joint enhances vector stability by eliminating internal genome rearrangements that occur during U_L_/U_S_ isomerization [38]. The joint deletion removes a region spanning positions from 116,982 to 132,605 of the HSV genome (nucleotide positions based on NC_001806). The remaining copy of ICP0 was then deleted, and an eGFP expression construct driven by the human cytomegalovirus (hCMV) major immediate early promoter was inserted in its place. The structure of the mutant virus was confirmed by PCR, sequencing, and Southern blot analysis.  Figure 1 shows the structure of the JD0G genome compared to that of wild-type HSV-1 KOS.

### 2.2. Glioblastoma Cell Lines Support Replication of the JD0G Virus

**Figure 2:**
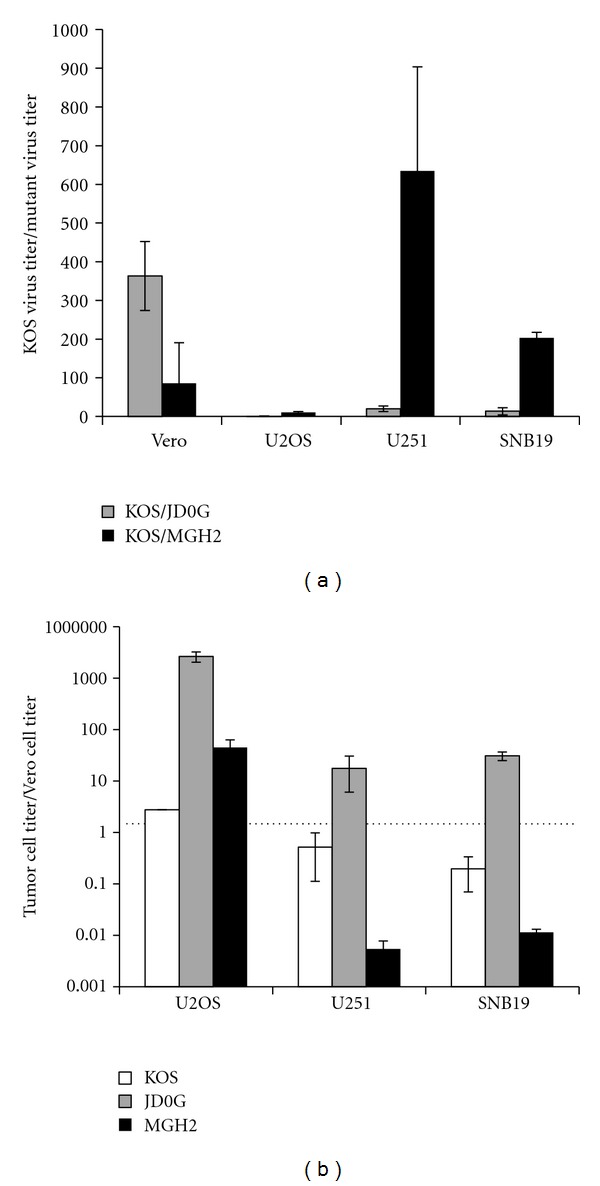
JD0G infection of glioblastoma cells results in efficient virus replication and tumor-selective cell death. (a) The replication of JD0G was evaluated in comparison to KOS and MGH2. HEL, U251, and SNB19 cell lines were infected in parallel with KOS, MGH2, or JD0G at an MOI of 0.1 based on U2OS titers. Supernatants were collected at 24 hpi, and the amount of virus produced was assessed by titration on U2OS cells. The data represent the average of three independent infections, and error bars represent the standard deviation. For all three viruses the differences in virus yield observed between HEL cells and either glioma cell line are statistically significant as determined by the student *t*-test (*P* values less than 0.03). (b) Tumor-specific cell death was assessed by counting the number of surviving cells following infection with either JD0G or MGH2 virus. HEL, U251 or SNB19 cells were infected at an MOI of 0.1, and Trypan Blue staining was performed to count the number of viable cells 48 h postinfection. The data represent the average of three independent infections, and error bars represent the standard deviation. All differences are statistically significant (*P* values less than 0.01).

Successful oncolytic virus treatment of GBM requires efficient, tumor-specific replication, and lysis of the tumor cell. We compared JD0G replication with that of wild-type KOS virus and another oncolytic vector, MGH2, a derivative of the well-studied vector G207 [32]. MGH2 lacks expression of *γ*34.5 and produces a nonfunctional ICP6 protein fused with GFP from the *U*_*L*_ *39* locus. In addition, MGH2 carries two genes that encode prodrug activating enzymes, *CYP2B1* (cytochrome p450), and *shiCE* (secreted human intestinal carboxylesterase). ICP0-deficient viruses are impaired for replication at low MOI in cells such as human embryonic lung fibroblasts (HEL cells), while certain tumor cell lines have been shown to allow efficient replication [22]. To assess the ability of human glioblastoma cell lines to support virus replication, HEL, SNB19, and U251 glioblastoma cells were infected at an MOI of 0.1 (based on U2OS titers), supernatants were collected at 24 hours postinfection (hpi) and virus yields were measured by titration on U2OS cells. Infections with KOS consistently generated slightly higher virus yields on HEL cells than on the two glioma cell lines (Figure 2(a)). In contrast, infection of the glioma cells with JD0G virus produced titers that were on average 10-fold higher than those obtained from HEL cells (Figure 2(a)). A similar comparison of MGH2 titers across the cell lines demonstrated that while MGH2 was able to replicate in HEL cells, its replication was substantially more impaired than that of JD0G on the two glioblastoma cell lines (Figure 2(a)). These results suggest that both glioblastoma cell lines provided an enhanced environment for JD0G growth, but not for MGH2, and that these tumor cells partially complement the ICP0 deficit.

We then examined the ability of the JD0G and MGH2 oncolytic viruses to cause tumor-specific cell death. HEL, SNB19, and U251 cells were infected at an MOI of 0.1, and the surviving cells were counted at 48 hpi (Figure 2(b)). Compared to mock-infected HEL cells, JD0G infection reduced the number of HEL cells by less than 2-fold. In contrast, the number of glioma cells that survived infection with JD0G was reduced by 10-fold or greater. MGH2 infection did not exhibit similar tumor-specific cell-killing activity, causing more cell death in the HEL cell line than in either glioma cell line. Thus, the JD0G oncolytic vector both exhibited a better tumor-selective replication profile than MGH2 and demonstrated clear glioma-specific cell death.

### 2.3. IFN*γ* Treatment Inhibits Virus Replication in an MOI-Dependent Manner

**Figure 3:**
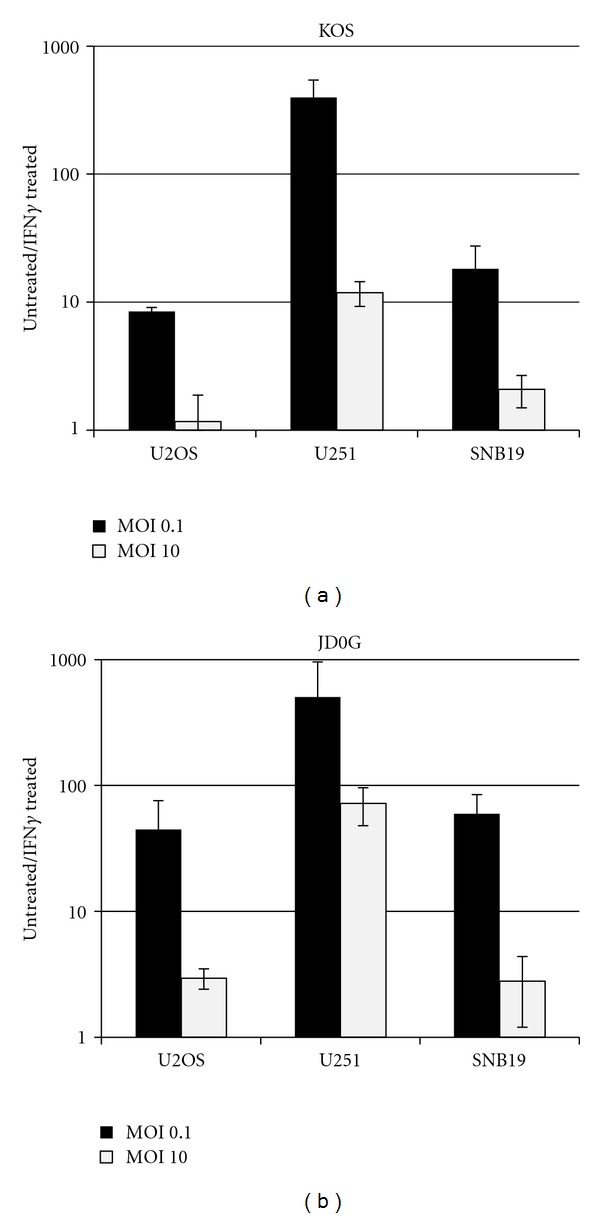
IFN*γ* treatment of glioblastoma cells results in a decrease in virus yield in an MOI-dependent manner. U251 and SNB19 cells were pretreated or mock treated with IFN*γ* (500 U/mL) 24 h prior to infection with either KOS or JD0G at an MOI of 0.1 (a) or 10 (b), and the supernatants were titered at 24 hpi. The data present the average of two independent infections, and the error bars represent the standard deviation; these data are representative of replicate experiments.

IFN*γ* presents a potential impediment to virus replication in tumor cells *in vivo*. To evaluate the sensitivity of KOS and JD0G to type II IFN, SNB19 and U251 cells were treated with IFN*γ* for 24 h and infected with either KOS or JD0G at an MOI of 0.1 or 10. Virus yields at 24 hpi were assessed by titration on U2OS cells. The results (Figures 3(a) and 3(b)) showed that (i) growth of KOS and JD0G was inhibited by IFN*γ* at low MOI on both cell lines; (ii) JD0G was somewhat more sensitive than KOS; (iii) both viruses at low MOI were hypersensitive to IFN*γ* on U251 cells; and (iv) high-MOI infection minimized IFN*γ* sensitivity. These results suggested that ICP0 deficiency has a moderate (≤5-fold) effect on IFN*γ* sensitivity at low MOI, a condition that likely models *in vivo* infection more realistically than high-MOI infection of cultured cells. In addition, the data indicated that both the antiviral response to IFN*γ* and the contribution of ICP0 to viral escape from this response at high MOI vary between glioblastoma lines.

### 2.4. IFN*γ*-Induced IDO Protein Has a Minimal Effect on Virus Replication

**Figure 4:**
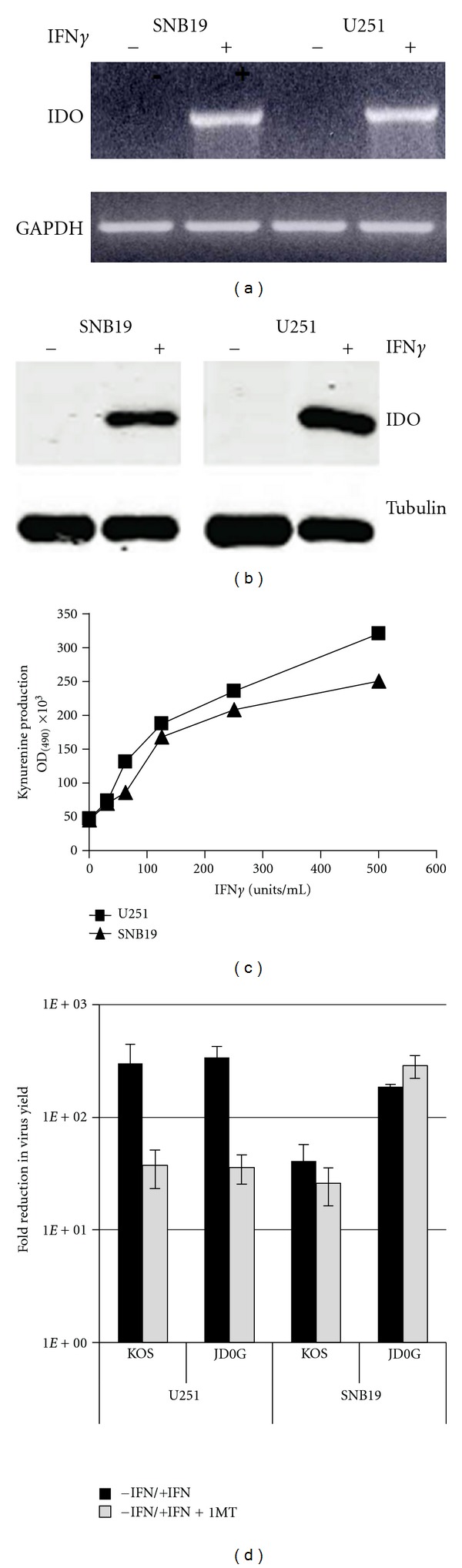
IFN*γ*-induced IDO protein has a minimal effect on virus replication. (a) SNB19 or U251 cells were pretreated with varying concentrations of IFN*γ* (0–500 U/mL) in media supplemented with 100 *μ*g/mL of L tryptophan. After 3 days of incubation, IDO activity was measured by determining kynurenine content in the cell supernatants. (b) U251 or SNB19 cell lines were pretreated or mock treated with IFN*γ* (500 U/mL) with or without 1-MT (300 *μ*M) 24 h prior to infection with JD0G or KOS virus, and the supernatants were titered at 24 hpi. The data represent the average of two independent infections, and the error bars represent the standard deviation; these data are representative of replicate experiments.

Local production of IFN*γ* induces the synthesis of indoleamine-2,3-dioxygenase (IDO), an enzyme that catalyzes degradation of the essential amino acid tryptophan producing kynurenine. IDO production in response to IFN*γ* has been linked to the inhibition of HSV replication in certain cell lines *in vitro *[27]. In order to determine if the glioblastoma cells used in this study produce active IDO protein either constitutively or in response to IFN*γ*, IDO-enzymatic activity was assessed by measuring the level of kynurenine in the supernatants of IFN*γ*-treated cells. Kynurenine levels increased in a dose-dependent manner in response to increasing levels of IFN*γ* (Figure 4(a)). These data illustrated that active IDO protein was produced in response to IFN*γ* treatment of glioblastoma cells.

We used the small molecule inhibitor of IDO activity, 1-methyl-tryptophan (1-MT), to assess the role of IDO in the observed reduction in virus yields from glioblastoma cells treated with IFN*γ* prior to low-MOI infection (Figure 4(b)). In U251 cells, IFN*γ* decreased both KOS and JD0G yields over 100-fold, and the addition of 1-MT consistently reduced this loss to approximately 15-fold; 1-MT alone had no effect on virus growth (data not shown). In contrast, the presence of 1-MT did not significantly alter the reduced yields of KOS and JD0G on IFN*γ*-treated SNB19 cells. These data suggested that IDO contributes to the IFN*γ* hypersensitivity of the two viruses on U251 cells, but not to their lower sensitivity on SNB19 cells. In both instances, however, the main effect of IFN*γ* appeared to be mediated via a different pathway.

### 2.5. Virus Infection Reduces the Amount of IDO mRNA and Protein in IFN*γ*-Treated Cells

**Figure 5:**
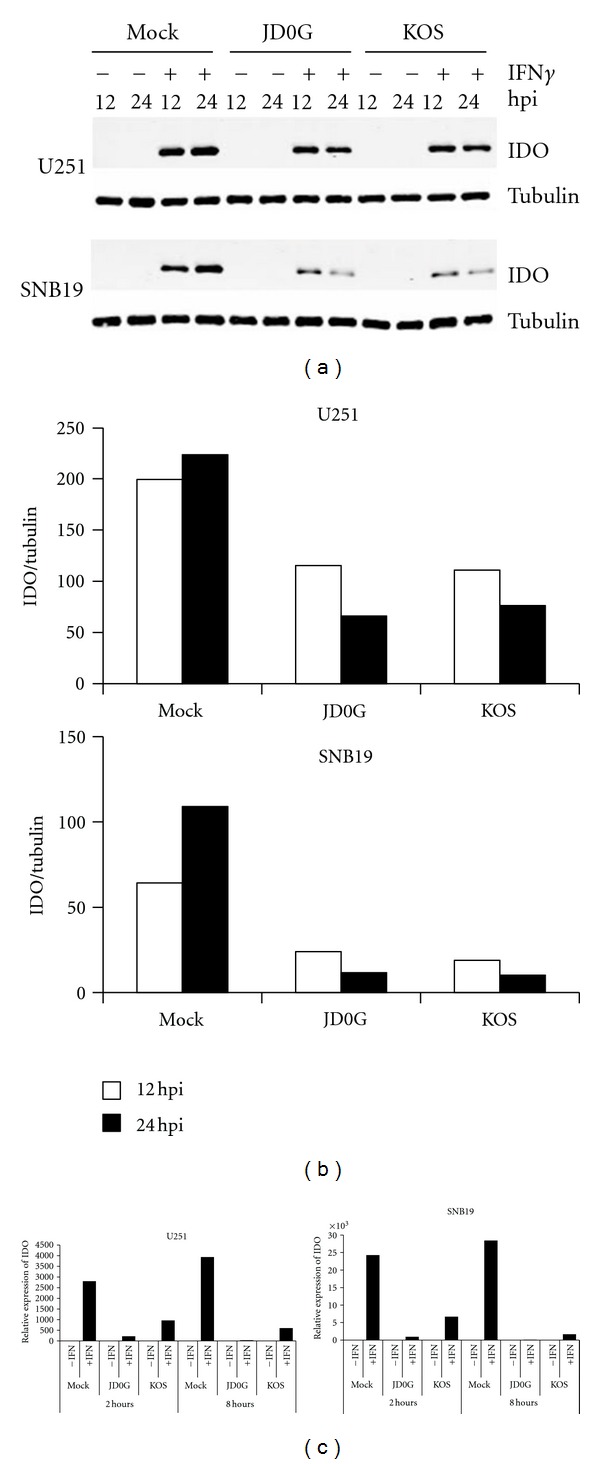
Reduction in IFN*γ*-induced IDO expression following virus infection of glioblastoma cells. (a) The effect of virus infection on IFN*γ*-induced IDO protein levels in SNB19 cells (top) and U251 cells (bottom) was visualized by Western blot. Cells were treated with IFN*γ* or mock treated for 24 h prior to infection with either JD0G or KOS virus. At 12 and 24 hpi, cell lysates were analyzed by Western blot with antibodies against IDO protein (upper panels) or *α*-tubulin (lower panels). (b) IDO protein levels were quantified using the Odyssey Infrared Imaging System, and the relative amount of IDO protein (IDO/*α*-tubulin) in each sample is shown. Data are representative of three experiments using different time points. (c) The effect of virus infection on IDO mRNA levels in SNB19 and U251 cells was assessed by quantitative RT-PCR. Cells were treated with IFN*γ* or mock treated for 24 h prior to infection with either JD0G or KOS virus, and total cellular RNA was collected at 2 and 8 hpi. IDO mRNA levels for each sample were normalized to GAPDH levels and the fold change in IDO expression for each sample is shown relative to the untreated, mock-infected control.

Although active IDO protein was produced in response to IFN*γ* in both U251 and SNB19 cells, the 1-MT experiments described above indicated that IDO activity did not significantly influence virus replication in at least one of these lines, SNB19. This may suggest that HSV-1 can counteract the IDO pathway to allow virus replication. The level of IDO protein present in U251 and SNB19 cells was measured following IFN*γ* treatment and virus infection (Figure 5(a)). Untreated cells and cells infected with virus alone did not show detectable levels of IDO protein at 12 and 24 hpi (Figure 5(a)). Cells treated with IFN*γ* produced IDO protein in both cell lines. However, cells treated with IFN*γ* and then infected with either KOS or JD0G virus showed a substantial reduction in the amount of IDO protein (Figures 5(a) and 5(b)). At 24 hpi, less than 20% of the IDO protein remained in SNB19 cells and less than 30% in U251 cells (Figure 5(b)). These data, representative of 3 independent experiments, suggested that infection of cells with HSV-1 can lead to downregulation of IDO protein.

Downregulation of IDO in response to virus infection can occur either at the protein or mRNA level. We therefore measured IDO mRNA expression by quantitative RT-PCR at 2 and 8 hours following virus infection of IFN*γ*-treated cells. The results (Figure 5(c)) demonstrated that IDO mRNA levels were reduced as early as 2 hours postinfection with either KOS or JD0G viruses when compared to mock-treated controls. Together these data suggest that infection of glioblastoma cells with HSV results in reduced expression of both IDO mRNA and protein, thereby minimizing the inhibitory effects of this protein on virus replication.

## 3. Discussion

The development of attenuated viruses that are adapted for preferential replication in solid tumors is an attractive approach to treatment of malignancies where more standard therapies are either ineffective or difficult to apply. HSV-1-oncolytic vectors used in early-phase clinical trials for the treatment of GBM have shown some success without serious side effects [33, 34]. Tumor specificity can be achieved by deleting viral genes that permit mutant virus replication in tumor cells while profoundly impairing virus replication in normal host cells [1]. The vector prototype is G207, that is, deleted for *γ*34.5 and produces a nonfunctional ICP6-LacZ fusion protein [35]. ICP6 encodes the large subunit of the viral ribonucleotide reductase, a protein that permits virus growth in nondividing cells by maintaining the nucleotide pool, while *γ*34.5 counteracts the virus-induced activation of the PKR pathway. The more advanced oncolytic vector examined in this study, MGH2, was derived from G207 by replacing *lacZ* with eGFP at the ICP6 locus and inserting two antitumor genes, *CYP2B1* (encoding cytochrome p450) and *shiCE* (encoding secreted human intestinal carboxylesterase) [32]. These enzymes activate the anticancer drugs cyclophosphamide and irinotecan, respectively, both of which are potent tumor toxic products. In one study, MGH2 showed oncolytic activity *in vivo* only with the addition of cyclophosphamide and irinotecan [32], indicating that the MGH2 vector backbone alone does not function as an effective OV. Some evidence suggests that vector replication in certain tumor cells may require *γ*34.5 activity and that oHSV is susceptible to innate immune responses, potentially limiting the effectiveness of this and other oHSV vectors [36]. We therefore sought to examine other mutant backbones that may overcome these limitations.

In this study we characterize an oHSV vector (JD0G) that is deleted for ICP0 and the joint elements of the viral genome. HSV-1 mutants defective for the production of ICP0 protein provide an attractive alternative to the MGH2 vector backbone. ICP0-deficient vectors are impaired for growth at low MOI in most cell lines, while replication preferentially occurs in certain tumor cell lines [22, 23, 37]. Moreover, Hummel and colleagues have reported that an HSV-1 double mutant lacking VP16 and ICP0 protein expression replicates efficiently in a variety of tumor cells derived from prostate, lung, colon, and mammary carcinomas with evidence of oncolytic activity in animal models [24]. Using the JD0G backbone, we sought to determine whether the unique interaction of an ICP0-deleted vector with tumor cells could be exploited to develop novel oncolytic viruses suitable for treatment of brain tumors. Our findings showed that JD0G replication was significantly enhanced in the U251 and SNB19 glioblastoma cell lines compared to HEL cells, while MGH2 replication was highly impaired, showing 2-3 logs lower yields on the glioblastoma lines than JD0G. These findings point to the possibility that genetic changes related to glioblastoma development relieve the need for ICP0 expression and permit JD0G replication, while the mutations in MGH2 are detrimental to vector growth and not complemented in these lines. Furthermore, the difference in replication between these two vectors was most pronounced at low multiplicity (data not shown), a condition that may be more relevant to circumstances *in vivo* than high-MOI infections in cell culture. An oHSV vector that can replicate efficiently from a small number of initial infectious particles is most likely to be effective in tumors.

It is likely that the tumor-specific replication of the JD0G vector can be ascribed to the ICP0 deficiency and not to the removal of the joint components of the viral genome. The HSV genome consists of two unique elements (U_L_ and U_S_) that are each flanked by repeated sequences (ab/b′a′; a′c′/ca) (Figure 1). This arrangement creates two terminal repeat regions that together contain one copy of the genes for ICP34.5, ICP4, ICP0, and the latency-associated transcript (LAT), and an internal repeat element (joint) that contains a second copy of each gene. Thus, the joint deletion generated in the construction of JD0G removes a single copy of each gene, reducing but not eliminating gene expression. This modification alone has a minimal effect on virus replication (unpublished observations, [38]). The benefit of this deletion is that it eliminates the possibility of genome isomerization events that typically occur between the repeated elements of the viral genome and generate four possible HSV isomers. Hence, the JD0G genome has enhanced stability over the wild-type KOS genome.

Following inoculation of oHSV* in vivo,* IFN*γ* can be produced by resident microglia, recruited macrophages, dendritic and NK cells at the growing tumor site [25, 39]. In response to IFN*γ*, dendritic cells and tumor cells may produce IDO, whose enzymatic activity leads to the degradation of tryptophan and the production of toxic catabolites such as kynurenine and quinolinate [40]. Additionally, tumor cells including glioblastoma cells have been reported to express IDO without IFN*γ* induction [41]. Tryptophan depletion can impede the production of viral proteins and, together with kynurenine, can induce effector T-cell anergy [30]. Therefore, IFN*γ* can negatively affect both oncolytic virus replication and tumor-specific immunity at several levels. Although IFN*γ*-mediated inhibition of oHSV replication may be overcome in part by the administration of immunosuppressive drugs such as cyclophosphamide, a drawback to this approach is that it will also inhibit the induction of tumor-specific immunity.

In view of these considerations, and to explore the tumor-related changes that may potentially influence the innate immune response to virus infection, KOS and JD0G viral replication in glioblastoma cells were tested for their sensitivity to IFN*γ* treatment. Our data demonstrate that both viruses were sensitive to IFN*γ* at low MOI, but the ability of IFN*γ* to control virus replication was largely lost at elevated MOI. However, we observed marked differences between the glioblastoma cell lines in both the IFN*γ* sensitivity of virus replication and the involvement of IDO activity in the inhibition of virus replication. SNB19 cells produced lower levels of IDO protein than U251 cells, and virus replication in SNB19 cells was less sensitive to IFN*γ* treatment. In the IFN*γ*-hypersensitive U251 cells, both KOS and JD0G were found to downregulate, but not eliminate, IDO mRNA and protein, and the IDO inhibitor 1-MT was able to improve the efficiency of virus replication. In contrast, both KOS and JD0G infection nearly eliminated IDO expression in IFN*γ*-treated SNB19 cells, and 1-MT did not improve virus replication. Together, these results indicate that IDO expression above a certain level can inhibit virus replication in glioblastoma cells, but also that the majority of IFN*γ*-induced inhibition of virus replication occurs via a different pathway. The limited role of IDO in controlling virus replication may be attributable to the virus-mediated downregulation of IDO observed on infection with both KOS and JD0G. This downregulation may not only allow for vector replication in the presence of IFN*γ*, but also minimize adverse effects on antitumor immunity. Thus, we propose that vector-induced IDO downregulation may be an important aspect of the therapeutic potential of oHSV vectors.

## 4. Methods

### 4.1. Cell Lines and Viruses

Human glioblastoma SNB19 and U251 [42], osteosarcoma U2OS (ATCC, Manassas, VA, USA), embryonic lung HEL (ATCCs), and monkey kidney Vero cells (ATCC) were cultured by standard methods. HEL cells were maintained in RPMI supplemented with 10% fetal bovine serum (FBS). All other cells were maintained in Dulbecco's Modified Eagle Medium (Atlanta Biologicals, Norcross, GA, USA) supplemented with 10% FBS at 37°C in 5% CO_2_. The wild-type HSV-1 (KOS) virus stock was prepared on Vero cells.

The JD0G mutant HSV-1 virus was derived by deletion of ICP0 by mixed infection/transfection of the Joint Deleted vector with plasmid 28E3. In order to obtain 28E.3 we initially deleted the fragment StuI/HpaI from the plasmid 28.1 (which consists of the Dra I fragment of psg28 [43], and a Bgl II linker was inserted into this site. Plasmid 28E3 was created by cloning a Bgl II fragment containing a HCMV eGFP expression construct from pEGFPN-1 (Clontech, Palo Alto, CA, USA) into the Bgl II site of 28B.

JD0G virus stocks were prepared on U2OS cells. The MGH2 virus was kindly provided by Antonio Chiocca (Ohio State University). For comparison purposes, MOIs were based throughout on viral titers determined on U2OS cells.

### 4.2. Virus Growth

1 × 10^5^ cells were seeded in 24-well dishes prior to infection. Confluent monolayers of cells were infected at an MOI of 0.1 in serum-free DMEM (Atlanta Biologicals) for 2 h. Supernatants were collected at 24 hpi, and viral titers were determined by serial dilution on U2OS cells. Plaques were visualized by crystal violet staining.

### 4.3. IFN*γ* Treatment

7 × 10^4^ cells were seeded in 24-well dishes and pretreated or mock treated for 24 h with 500 U/mL of recombinant human IFN*γ* (Cell Sciences, Canton, MA, USA). The IDO inhibitor 1-methyl-L-tryptophan (Sigma, St. Louis, MO, USA) was used at a final concentration of 300 *μ*M. Pretreated cells were infected at the appropriate MOI in serum-free DMEM for 2 h, virus was removed, and the cells were fed with fresh media plus IFN*γ*. Supernatants were collected at 24 hpi and viral titers were determined by serial dilution on U2OS cells. Plaques were visualized by crystal violet staining.

### 4.4. Quantitative RT-PCR

3 × 10^5^ cells were seeded in 6-well dishes and pretreated or mock treated for 20 h with 500 U/mL of IFN*γ*. Pretreated cells were infected at an MOI of 20 in serum-free DMEM with either KOS or JD0G for 2 h, virus was removed, and fresh media with IFN*γ* was added to the wells. Cells were harvested at the indicated times postinfection (either 2 or 8 h), and total RNA was extracted using the RNeasy kit (QIAGEN, Valencia, CA, USA). 2 *μ*g of total RNA was used to synthesize the first cDNA strand using Superscript First-Strand Synthesis System (Invitrogen, Carlsbad, CA, USA). Quantitative PCR was performed as previously described [44]. Average *C*_T_ values for IDO were normalized with average *C*_T_ values for GAPDH, and data were plotted as the fold change relative to untreated, mock-infected controls using the 2^−ΔΔ*C*_T_^ method [45]. The following primers and probes were obtained from PE Applied Biosystems (Foster City, CA, USA): IDO Fwd, GGTCATGGAGATGTCCGTAA; IDO Rev, ACCAATAGAGAGACCAGGAAGAA; IDO Probe, 6FAM-CTGTTCCTTACTGCCAACTCTCCAAGAAACTG-Tamra; GAPDH-FWD, CCCCACACACATGCACTTACC; GAPDH-REV, CCTACTCCCAGGGCTTTGATT; GAPDH-Claw-Probe, 6FAM-AAAGAGCTAGGAAGGACAGGCAACTTGGC-TAMRA.

### 4.5. Kynurenine Assay

3 × 10^4^ cells were pretreated with varying concentrations of IFN*γ* (0–500 U/mL) in media supplemented with 100 *μ*g/mL L-tryptophan. After 3 days of incubation, supernatants were harvested for determination of kynurenine content. Samples of 160 *μ*L were incubated with 10 *μ*L of 30% TCA for 30 min at 50°C and centrifuged at 3,000 rpm for 20 min at room temperature. 100 *μ*L of each sample was reacted with an equal volume of Ehrlich reagent (Sigma) at room temperature for 10 min, and the OD_490_ measured using a microplate reader.

### 4.6. Western Blot

3 × 10^5^ cells were seeded in 6-well dishes and pretreated or mock treated for 20 h with 500 U/mL of IFN*γ*. Pretreated cells were infected at an MOI of 20 in serum-free DMEM with either KOS or JD0G for 2 h, virus was removed, and fresh media with IFN*γ* was added to the wells. Cells were harvested at the indicated times postinfection (either 12, or 24 h), resuspended in 200 *μ*L lysis buffer (10 mM Tris pH 7.4, 150 mM NaCl, 5 mM EDTA pH 8, 0.1% Triton-X, 1 mM DTT, 1 mM PMSF, 1 mM NaVO_4_, and 0.2% Protease Inhibitor Cocktail [Sigma]), and incubated on ice for 30 min. 5 *μ*g of total protein was resolved on an 8% SDS-PAGE gel and transferred to Protran 0.45 *μ*m nitrocellulose membrane (Whatman, Piscataway, NJ, USA). The membrane was blocked for 1 h at room temperature in Tween-20-free Odyssey blocking buffer (LI-Cor Biosciences, Lincoln, NE, USA) and incubated overnight at 4°C with a 1 : 5,000 dilution of ALX-210-429 rabbit anti-IDO antibody (Alexis Biochemicals, Lausanne, Switzerland) and a 1 : 20,000 dilution of mouse anti-*α*-tubulin antibody (T6074, Sigma) in 1x PBS/0.05% Tween-20. The membrane was washed with 1x PBS/0.05% Tween-20, incubated for 1 h at room temperature with goat anti-mouse IRDye800CW-green and goat anti-rabbit IRDye680-red secondary antibodies (both from LI-Cor), and washed with 1x PBS/0.05% Tween-20. The membrane was scanned with Odyssey Infrared Imaging System (LI-Cor) and analyzed with Odyssey 3.0 software as specified by the manufacturer.

### 4.7. Cell Killing

1 × 10^6^ cells were seeded in 6-well dishes and infected 24 hours later at an MOI of 0.1 in serum-free DMEM (Atlanta Biologicals). Media was replaced with fresh serum-containing media after 1.5 h and 48 h postinfection viable cells were stained with Trypan Blue and counted.
